# An Index of Diseases and Their Treatment

**Published:** 1877-10

**Authors:** 


### BIBLIOGRAPHICAL.

*An Index of Diseases and their Treatment.*—By Thomas Hawkes Tanner, M. D., F. R. C. P., Etc. Second Edition Revised by W. H. Broadbent, M. D., F. R. C. P., Etc. Publishers: Lindsay and Blackiston, Philadelphia.

This work is so arranged as to present a brief, but comprehensive, and classified description of diseases and therapeutics, and must prove a useful adjunct to the more voluminous treatise. Under some sixty general divisions, are included nearly one thousand diseases, a brief description of the symptoms and treatment of each being given, which renders the work a great assistant to the medical practitioner. An appendix of formulæ occupies some one hundred and fifty pages, describing the preparation of Aliments, the composition of Alteratives and Resolvents, Antacids, Astringents, and all other articles of the materia medica in general use, ending with Climates for Invalids, and Mineral Waters.

While the purely medical affections are correctly treated, the same cannot be said of the dental, as the treatment of odontalgia recommended is by no means such as an educated dental practitioner would pursue. The author, however, is not behind the majority of his medical brethren in this respect, for many of the most prominent are sadly wanting in their knowledge of the dental organs, at least so far as their diseases are concerned.

